# Tumor-infiltrating immune cell types predicting recurrence-free survival in melanoma patients receiving adjuvant PD-1 inhibitor therapy

**DOI:** 10.3389/fimmu.2026.1886388

**Published:** 2026-07-31

**Authors:** Andrea Ladányi, Georgina Fröhlich, Barbara Hegyi, Patrik Horváth, Tímea Balatoni

**Affiliations:** 1 Department of Surgical and Molecular Pathology, National Institute of Oncology, Budapest, Hungary; 2 National Tumor Biology Laboratory, National Institute of Oncology, Budapest, Hungary; 3 Center of Radiotherapy, National Institute of Oncology, Budapest, Hungary; 4 Department of Biophysics, Eötvös Loránd University, Budapest, Hungary; 5 Department of Thoracic and Abdominal Tumors and Clinical Pharmacology, National Institute of Oncology, Budapest, Hungary; 6 Department of Oncodermatology, National Institute of Oncology, Budapest, Hungary

**Keywords:** adjuvant, biomarker, immunotherapy, melanoma, PD-1 inhibitors, tumor-infiltrating immune cells

## Abstract

**Background:**

Adjuvant treatment of melanoma patients with PD-1-based immunotherapy has improved recurrence rate, and became a standard treatment. However, a considerable proportion of patients recur within 1–2 years, necessitating the identification of predictive markers. Our study aimed to examine the association of intratumoral infiltration by specific immune cell subsets with the recurrence-free survival of melanoma patients receiving adjuvant PD-1 inhibitor therapy.

**Materials and methods:**

Archived paraffin blocks of pretreatment surgical samples from 48 melanoma patients receiving adjuvant PD-1 inhibitor therapy were selected. Intratumoral density of immune cells expressing the following markers: CD8, FOXP3, CD20, CD103, CD134, PD-1, and PD-L1 was determined by immunohistochemistry, and the associations with recurrence-free survival were analyzed.

**Results:**

Eighteen of the 48 patients developed recurrence during the follow-up period. In this group the ratio of patients showing strong immune cell infiltration (higher than the cutoff values defined by ROC curve analysis) was significantly lower compared to recurrence-free patients in the case of CD8^+^ T cells (2/18 vs. 16/30, p=0.0050), CD103^+^ tissue resident T cells (2/18 vs. 13/30, p=0.0259), and the activation/immune checkpoint markers CD134 (4/17 vs. 21/30, p=0.0029) and PD-1 (2/18 vs. 14/30, p=0.0134), while the evaluation of FOXP3^+^ cells yielded near significant trend (8/18 vs. 22/30, p=0.0663). High intratumoral density of the above cell types was accompanied with significantly longer recurrence-free survival.

**Conclusions:**

Our findings indicate the importance of immune cell infiltration in the efficacy of adjuvant PD-1 inhibitor treatment in a “real world” patient cohort.

## Introduction

Immune checkpoint inhibitors (ICIs) targeting the PD-1/PD-L1 pathway have markedly improved the management of many cancers, proving highly efficient in melanoma in the metastatic, adjuvant and neoadjuvant settings as well (1, 2). In patients with high-risk, resectable stage III/IV melanoma, adjuvant PD-1 inhibitors (nivolumab, pembrolizumab) significantly reduced the recurrence rate and enhanced recurrence-free survival (RFS), therefore becoming a mainstay of treatment of this patient population (1–3). Regardless, a significant proportion of patients still experience recurrence (25-30% within one year; the 5-year RFS is approximately 50%) (4–6). Therefore, reliable predictive biomarkers reflecting real-world data are needed to enable identification of patients who would require alternative treatment strategies.

In melanoma patients PD-1 inhibitors are also broadly used in the metastatic setting, for which several potential predictive markers have been proposed. These include, among others, PD-L1 expression, tumor mutational burden (TMB) or neoantigen load, tumor cell MHC expression, immune cell infiltration and immune-related gene expression (7–13). However, fewer results are available concerning predictive and/or prognostic biomarkers in the case of adjuvant immunotherapy (6, 14, 15). The most consistent finding was the association of the density of CD8^+^ T-cells (or some subpopulations) with RFS, while TMB and PD-L1 expression was also found to predict disease outcome in some studies (6, 14–16).

The above biomarker suggestions were mainly based on results obtained in the course of clinical trials (6, 15). The aim of our study was to evaluate the potential of a panel of immune cell types infiltrating metastatic tumors to predict recurrence probability in a real-world cohort of melanoma patients receiving adjuvant PD-1 inhibitor therapy. Among immune cell types, we focused on those that proved to be associated with response to PD-1 inhibitor treatment in our previous study on melanoma patients receiving anti-PD-1 therapy in metastatic setting (12). The markers included CD8 (cytotoxic T lymphocytes), FOXP3 (regulatory T cells), CD103 (marker of tissue-resident T lymphocytes), CD20 (B cells), CD134 (OX40; T-cell activation marker and costimulatory receptor, member of the TNF receptor superfamily), the PD-1 immune checkpoint receptor and its ligand, PD-L1.

## Materials and methods

### Patients and tumor samples

Archived paraffin blocks of pretreatment surgical samples (71 lymph node and 12 skin/s.c. metastases) from 48 melanoma patients receiving adjuvant PD-1 inhibitor therapy (nivolumab 240 mg every two weeks or 480 mg every four weeks; pembrolizumab 200 mg every 3 weeks or 400 mg every 6 weeks, for up to one year, start of treatment in 2019–2023) at the National Institute of Oncology were selected (**Table 1**). Only patients receiving at least 3 cycles of anti-PD-1 therapy were included. Sample collection was restricted to metastases surgically removed within one year before start of adjuvant therapy. Patients’ follow-up was performed by CT (and also by MRI or PET/CT if necessary) every 3 months. Recurrence-free survival (RFS) was defined as the time from commencing anti-PD-1 treatment to disease recurrence or death, or last follow-up. The median follow-up time was 56 months (14–80 months).

**Table 1:** Patient and sample characteristics.

Parameters	No relapse n=30	Relapse n=18	P value
Age			0.7723
≤60 years	12	8	
>60 years	18	10	
Gender			0.5897
female	11	9	
male	19	9	
ECOG performance status			0.6584
0	27	15	
1	3	3	
Stage			0.3431^a^
IIIB	12	4	
IIIC	15	9	
IIID	1	2	
IV	2	3	
BRAF mutation status			0.3454^b^
wild type	21	11	
mutant	7	7	
ND	2	0	
LDH level			1.0000
normal	29	17	
>ULN	1	1	
Anti-PD-1 drug			0.0681
nivolumab	9	11	
pembrolizumab	21	7	

^a^ Stage IIIB vs. all other stages combined.

^b^ wild type vs. mutant.

ECOG, Eastern Cooperative Oncology Group; ND, not determined; ULN, upper limit of normal.

### Immunohistochemical staining and evaluation

Immunohistochemical staining of tissue sections of formalin-fixed, paraffin-embedded tumor samples was performed using standard methodology as described earlier (12, 17). Briefly, after blocking endogenous peroxidases in deparaffinized sections by 3% H_2_O_2_ in methanol, antigen retrieval was performed by heating at 98°C for 40 min in citrate buffer (pH 6.0), followed by incubation with protein blocking solution (Protein Block, Serum-Free, Dako) for 10 min at room temperature. The following primary antibodies were applied (overnight incubation at 4°C): CD8 (C8/144B; Dako, Glostrup, Denmark), CD20y (L26; Dako), FOXP3 (236/E7; eBioscience, San Diego, CA), CD103 (EPR4166 (2); Abcam, Cambridge, UK), CD134 (Ber-ACT35; BioLegend, San Diego, CA), PD-1 (NAT-105; Bio SB, Santa Barbara, CA), PD-L1 (73-10; Abcam). Staining was detected using the EnVision+ System HRP Labelled Polymer Anti-mouse reagent (Dako) and the MACH2 Rabbit HRP-Polymer (Biocare Medical, Pacheco, CA) for mouse and rabbit primary antibodies, respectively, followed by staining with 3-amino-9-ethylcarbazole (AEC; Vector Laboratories, Inc., Burlingame, CA) and hematoxylin counterstaining.

Evaluation of immunohistochemical staining was performed as described earlier (12, 17), by light microscope equipped with an eyepiece graticule, independently by two researchers who were blinded to the clinical information, and the mean value of their separate counts was used for the analysis. Inter-observer agreement in density values was excellent (correlation coefficients between 0.7861 and 0.9785 for the different immune cell markers). The expression of PD-L1, similarly to other markers, was evaluated on immune cells only. Labeled cells within the metastases were counted in at least 10 randomly chosen fields per section, using a graticule of 10×10 squares designating an area of 0.0625 mm^2^ at 400× magnification. For patients with more than one metastasis available the average scores were calculated for each marker. Cutoff levels were set up based on ROC curve analysis, and the proportion of patients with a mean cell density higher than the cutoff level was also determined.

### Statistical analysis

Differences in the proportion of cases between groups were analyzed with Fisher’s exact test, using the Benjamini–Hochberg procedure for controlling the false discovery rate of multiple testing. The Kaplan–Meier analysis with log-rank test was applied for calculating differences in RFS. Univariate and multivariate analysis of RFS was also performed using Cox proportional hazards model. Differences were considered significant in the case of p values ≤ 0.05. Statistics were calculated using the Statistica software version 12.5 (StatSoft, Tulsa, OK, USA).

## Results

Eighteen of the 48 melanoma patients involved in this study developed recurrence during the follow-up period. None of the clinical parameters (**Table 1**) showed significant association with recurrence, although a trend could be observed for higher prevalence of patients treated with pembrolizumab in the subgroup not exhibiting relapse (p=0.0681).

**Figure 1:**
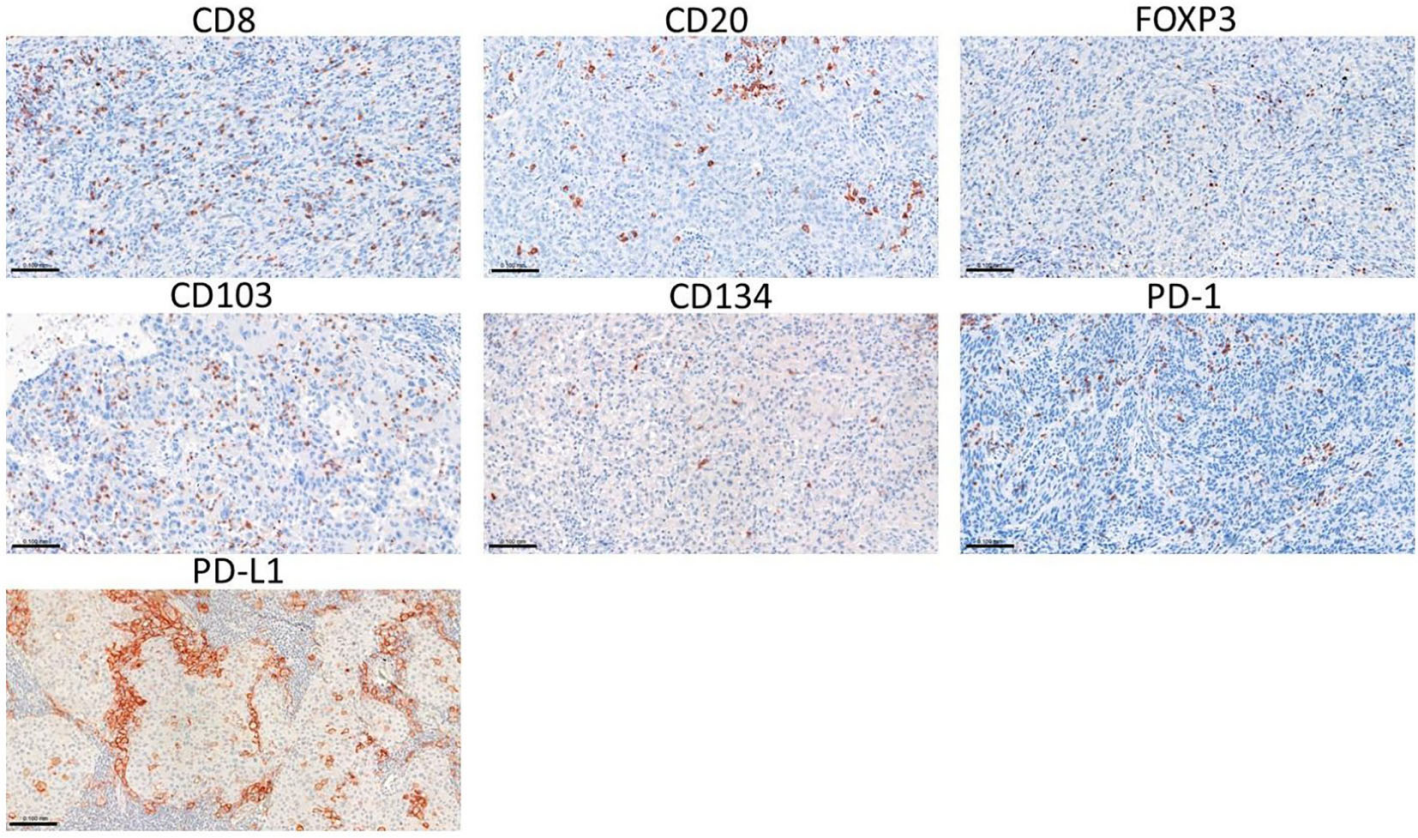
Immunohistochemical labeling of immune cell markers in metastatic melanoma samples (AEC, red; scale bar: 100 μm).

Using immunohistochemistry, we determined the intratumoral density of immune cells expressing the following markers: CD8, FOXP3, CD20, CD103, CD134, PD-1, and PD-L1 (**Figure 1**; **Supplementary Figure 1**). Of the immune cell types studied, CD8^+^ T lymphocytes were the most numerous, followed by PD-1^+^ and CD103^+^ cells, while CD134^+^ lymphocytes were present in the lowest number. Analyzing the proportion of patients with high mean intratumoral cell density (greater than the defined cutoff levels) showed higher prevalence in recurrence-free patients in the case of CD8^+^ T lymphocytes, CD103^+^ tissue resident T cells, and the activation/immune checkpoint markers CD134 and PD-1, while a near significant trend was observed in the case of FOXP3^+^ cells (**Table 2**). All associations with p values <0.05 remained significant when using the Benjamini–Hochberg procedure for controlling the false discovery rate of multiple testing.

**Table 2:** Relationship of relapse with the density of immune cells infiltrating metastases.

Proportion of cases with high cell density (%)			
Immune cell marker (cutoff)	No relapse n=30	Relapse n=18	P value
CD8 (>300.8 cells/mm^2^)	16/30 (53%)	2/18 (11%)	**0.0050**
CD20 (>44.8 cells/mm^2^)	13/30 (43%)	4/18 (22%)	0.2137
FOXP3 (>32.0 cells/mm^2^)	22/30 (73%)	8/18 (44%)	0.0663
CD103 (>144.0 cells/mm^2^)	13/30 (43%)	2/18 (11%)	**0.0259**
CD134 (>3.04 cells/mm^2^)	21/30 (70%)	4/17^a^ (24%)	**0.0029**
PD-1 (>140.8 cells/mm^2^)	14/30 (47%)	2/18 (11%)	**0.0134**
PD-L1 (>25.6 cells/mm^2^)	19/29^b^ (66%)	8/18 (44%)	0.2264

^a,b^ One case each for CD134 and PD-L1 staining could not be evaluated due to technical reasons. Bold p values indicate significant differences.

Restricting the analysis only to lymph node metastatic samples (yielding lower case number, 29 non-relapsing and 16 relapsing patients) yielded similar trends, with retaining significance in the case of CD8 and CD134 (results not shown).

The densities of most of the studied immune cell types strongly correlated with each other and they frequently showed coordinate presence. High expression of at least 4 of the 7 markers studied was found in 18 of 30 recurrence-free cases (60%), compared to only 2 of 18 patients showing recurrence (11%) (p=0.0010).

**Figure 2:**
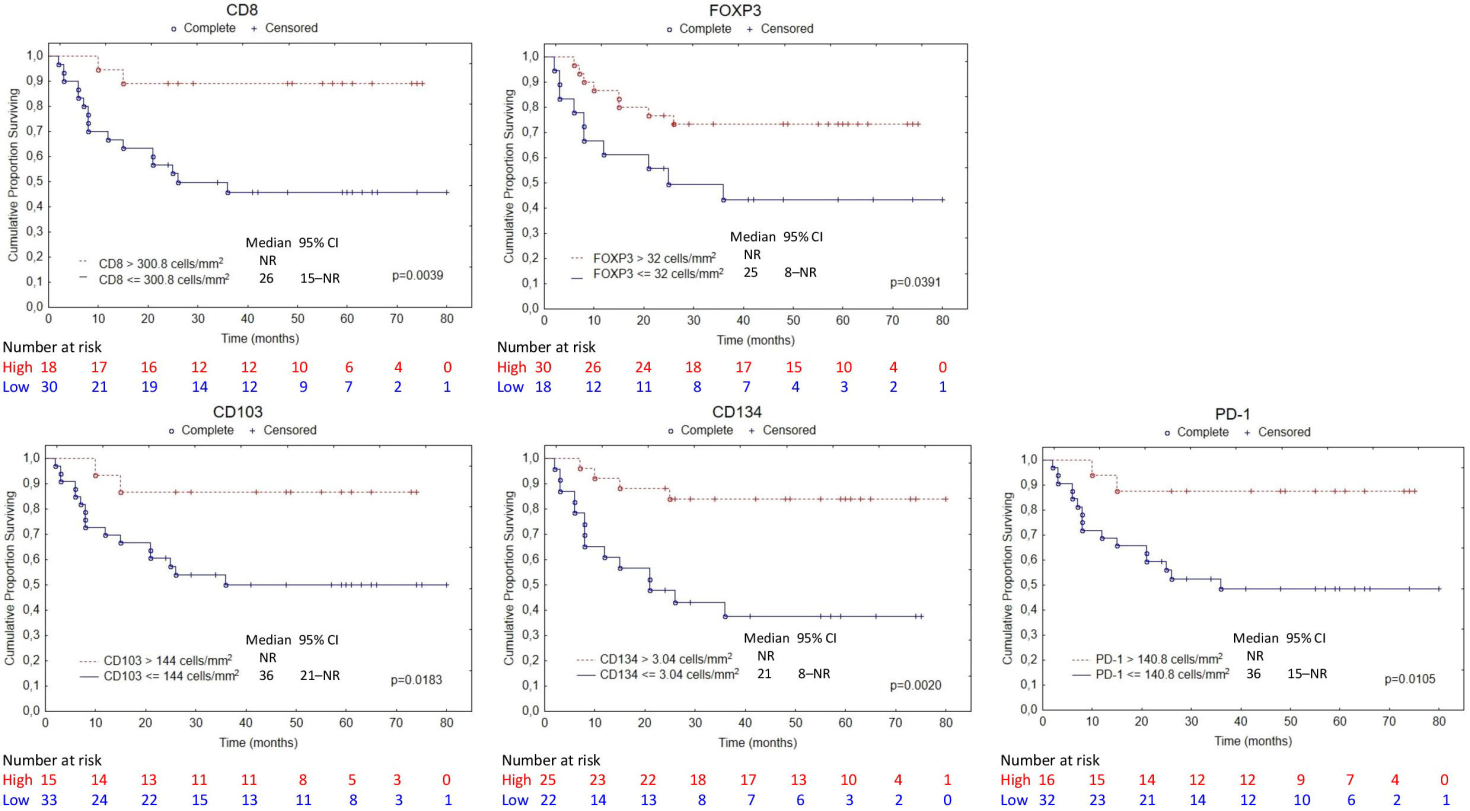
Kaplan-Meier curves of relapse-free survival of melanoma patients subdivided according to the intratumoral density of CD8^+^, FOXP3^+^, CD103^+^, CD134^+^, and PD-1^+^ immune cells.

Kaplan-Meier analysis of RFS revealed significant difference in the case of 5 cell types: CD8^+^, FOXP3^+^, CD103^+^, PD-1^+^, CD134^+^, all showing longer RFS of patients with high immune cell density in pretreatment metastases (p=0.0039, p=0.0391, p=0.0183, p=0.0105, and p=0.0020, respectively; **Figure 2**). Univariate analysis of RFS using Cox proportional hazards model revealed significant association with density values of the same cell types showing significant association according to Kaplan-Meier analysis, while among clinicopathological parameters, only the anti-PD-1 antibody used proved significant, favoring pembrolizumab (**Supplementary Table 1**). None of the above parameters proved significant in multivariate analysis including the variables that were found significant in univariate analysis; CD134^+^ cell density demonstrated near significant result (p=0.0788).

## Discussion

Adjuvant therapy of high-risk melanoma patients with PD-1 inhibitors proved efficient in improving recurrence-free survival, however, recurrence still occurs in a considerable proportion of cases. While a plethora of potential biomarkers have been investigated for predictive effect of immunotherapy in the metastatic setting, few results are available with regard to markers that could be applied in the clinic for improving patient selection in the case of adjuvant treatment.

In this study we investigated the predictive/prognostic potential of immune cell types infiltrating melanoma metastases, applying immunohistochemistry with a panel of 7 markers. We observed significant association between higher proportion of patients with pronounced immune cell infiltration and lack of recurrence and/or longer RFS in the case of 5 markers: CD8, CD103, PD-1, FOXP3, CD134. Our results concerning the value of CD8^+^ lymphocytes predicting recurrence probability corroborate earlier findings consistently showing increased prevalence in recurrence-free cases in melanoma patients receiving adjuvant ICI therapy (6, 14, 15). Furthermore, Attrill et al. studied CD8^+^ cell subpopulations and found a reduced recurrence risk in patients with high proportion of CD39^+^CD103^+^PD-1^+^ cells, but similar association was observed in the case of the CD39^-^CD103^+^PD-1^+^ T-cell subpopulation, so the phenomenon seems to apply for all CD103^+^ and PD-1^+^ T cells regardless of CD39 expression (14). CD103 is a marker of tissue-resident (or tumor-resident) memory T cells (TRM), also expressing other markers as CD69 and PD-1 (18, 19). These cells have been shown to accumulate in tumors of immunotherapy responders, and it has been proposed that they are the principal mediators of the therapeutic efficacy of ICIs (19–23). Co-expression of CD39 and CD103 was demonstrated to identify tumor-reactive CD8^+^ T lymphocytes in several human tumor types (24); earlier, the enrichment of tumor reactivity in CD8^+^ T cells expressing CD103 or PD-1 has also been described (25, 26). In our present study we did not investigate the co-expression of CD103 and PD-1, but both of them were found predictive of disease outcome, and their expression showed very strong correlation, similarly to our earlier study on metastatic melanoma patients treated with PD-1 inhibitors (12); this may suggest that the majority of T lymphocytes expressing these markers could be double positive TRM-like cells.

In contrast to PD-1, PD-L1 expression by immune cells did not prove predictive of recurrence, unlike our findings in the metastatic setting where it was found associated with clinical response to PD-1 inhibitors (12). Few studies investigated immune cell PD-L1 expression with regard to predictivity of the effect of adjuvant PD-1 blocking in melanoma patients. In a recent study evaluating the predictive/prognostic value of PD-L1 expression in a tissue-specific way, PD-L1 expression on immune cells in the primary tumor, but not in metastases, was found relevant in predicting RFS (16).

The density of FOXP3^+^ cells showed a non-significant trend for association with lack of recurrence, and a significant but moderately strong association with RFS. Higher intratumoral FOXP3^+^ cell densities were also reported in responders to anti-PD-1 and/or anti-CTLA-4 treatment of metastatic melanoma patients (10, 12, 17), which may reflect the upregulation of immunosuppressive mechanisms in a tumor microenvironment showing high immune activity, probably as a counter-regulatory mechanism. A strong positive correlation (p<0.001) between CD8^+^ T lymphocytes and FOXP3^+^ cell density was observed in the present study as well as in previous investigations (17, 27), and it has been proved in murine models that the presence of inhibitory mechanisms (including FOXP3^+^ regulatory T cells) was driven by infiltrating CD8^+^ T lymphocytes (27).

According to our results, the density of CD134^+^ cells proved to have the strongest predictive/prognostic value, demonstrating the lowest p values in our analyses. To the best of our knowledge, this study is the first to associate CD134^+^ cell infiltration with recurrence-free survival after adjuvant anti-PD-1 treatment in melanoma patients. In our earlier studies, expression of the T-cell activation marker/costimulatory molecule CD134 (OX40) on tumor-infiltrating T cells also proved a biomarker predicting response to anti-CTLA-4 as well as anti-PD-1 therapy in metastatic melanoma patients (12, 17).

Major strengths of our study are that it was performed on a ‘real world’ patient cohort, and we used whole sections from surgical samples, from more than one metastasis per patient if available, reducing the impact of intra- and inter-lesional heterogeneity. On the other hand, we recognize the inherent limitation of the study caused by its retrospective nature. Furthermore, the case number was constrained by the selection criteria we applied in order to decrease patient and sample variability (including only lymph node and skin/s.c. metastases, removed within one year before the start of adjuvant immunotherapy). Moreover, although no significant difference was found in the distribution of relapsing and not relapsing cases according to the clinicopathological parameters studied, there was a non-significant trend in the case of the applied drugs (nivolumab vs. pembrolizumab), and the potential confounding effects could not be addressed using stratified analyses because of the low number of cases in the subgroups. Our study applied single-marker immunohistochemistry, which cannot identify specific immune cell subpopulations defined by co-expression of certain markers, and did not aim the comprehensive characterization of the tumor immune microenvironment; its main objective was to suggest predictive markers that could be determined using widely available techniques, such as IHC, which can be applied in routine clinical practice. Finally, similarly to the majority of previous studies aiming at characterizing local immune parameters predicting the outcome of adjuvant PD-1 inhibitor therapy in melanoma patients, the lack of untreated control group limited the ability to distinguish predictive and prognostic biomarkers. Determining whether the resulted markers are specific for anti-PD-1 therapy (rather than simply reflecting a more favorable baseline antitumor immune response associated with improved outcomes regardless of treatment) would need further comparative assessment.

In conclusion, our data suggest that infiltration by CD8^+^ T lymphocytes, CD103^+^ tissue resident T cells, and lymphocytes expressing the activation markers/immune checkpoints PD-1 and CD134 could be considered as candidate biomarkers predicting recurrence in melanoma patients receiving adjuvant PD-1 inhibitor therapy. Our study provided the first evidence for the predictive value of CD134^+^ cells in this setting, which warrants validation on larger prospective patient cohorts.

## Author's note

Part of this work was presented as poster at EACR 2025, June 16-19, 2025, Lisbon, Portugal (28).

## Data Availability

The raw data supporting the conclusions of this article will be made available by the authors, without undue reservation.
