# Supplementary material for: Tumor-infiltrating immune cell types predicting recurrence-free survival in melanoma patients receiving adjuvant PD-1 inhibitor therapy
Source: Front Immunol. 2026 Jul 31;17:1886388. doi: 10.3389/fimmu.2026.1886388 (PMC13473003; doi:10.3389/fimmu.2026.1886388)
Supplement: Supplementary file 1 [file DataSheet1.pdf]

Supplementary Table 1. Univariable and multivariable Cox regression of recurrence-free survival

	Univariable		Multivariable	
**Variable**	**HR (95% CI)**	**p value**	**HR (95% CI)**	**p value**
**Age**		0.6931		
≤60 years	1			
>60 years	0.83 (0.33–2.10)			
**Gender**		0.5524		
female	1			
male	0.76 (0.30–1.90)			
**ECOG performance status**		0.6146		
0	1			
1	1.38 (0.40–4.76)			
**Stage**		0.2411		
IIIB	1			
IIIC/IIID/IV	1.95 (0.64–5.92)			
**BRAF mutation status**		0.3387		
wild type	1			
mutant	1.59 (0.62–4.10)			
**LDH level**		0.6873		
normal	1			
>ULN	1.51 (0.20–11.41)			
**Anti-PD-1 drug**		**0.0246**		0.2206
nivolumab	1		1	
pembrolizumab	0.33 (0.13–0.87		0.52 (0.18–1.48)	
**CD8^{+} cells**		**0.0148**		
≤300.8 cells/mm^{2}	1		1	0.1536
>300.8 cells/mm^{2}	0.16 (0.04–0.70)		0.13 (0.08–2.15)	
**CD20^{+} cells**		0.079		
≤44.8 cells/mm^{2}	1			
>44.8 cells/mm^{2}	0.37 (0.12–1.12)			
**FOXP3^{+} cells**		**0.0420**		0.6373
≤32.0 cells/mm^{2}	1		1	
>32.0 cells/mm^{2}	0.38 (0.15–0.97)		1.29 (0.44–3.75)	
**CD103^{+} cells**		**0.0409**		0.6393
≤144.0 cells/mm^{2}	1		1	
>144.0 cells/mm^{2}	0.22 (0.05–0.94)		0.51 (0.03–8.67)	
**CD134^{+} cells**		**0.0048**		0.0788
≤3.04 cells/mm^{2}	1		1	
>3.04 cells/mm^{2}	0.20 (0.06–0.61)		0.31 (0.08–1.15)	
**PD-1^{+} cells**		**0.0282**		0.9963
≤140.8 cells/mm^{2}	1		1	
>140.8 cells/mm^{2}	0.19 (0.04–0.84)		1.01 (0.04–28.59)	
**PD-L1^{+} cells**		0.1068		
≤25.6 cells/mm^{2}	1			
>25.6 cells/mm^{2}	0.46 (0.18–1.18)			
