# Supplementary material for: Tumor-infiltrating immune cell types predicting recurrence-free survival in melanoma patients receiving adjuvant PD-1 inhibitor therapy
Source: Front Immunol. 2026 Jul 31;17:1886388. doi: 10.3389/fimmu.2026.1886388 (PMC13473003; doi:10.3389/fimmu.2026.1886388)
Supplement: Supplementary file 2 [file DataSheet2.pdf]

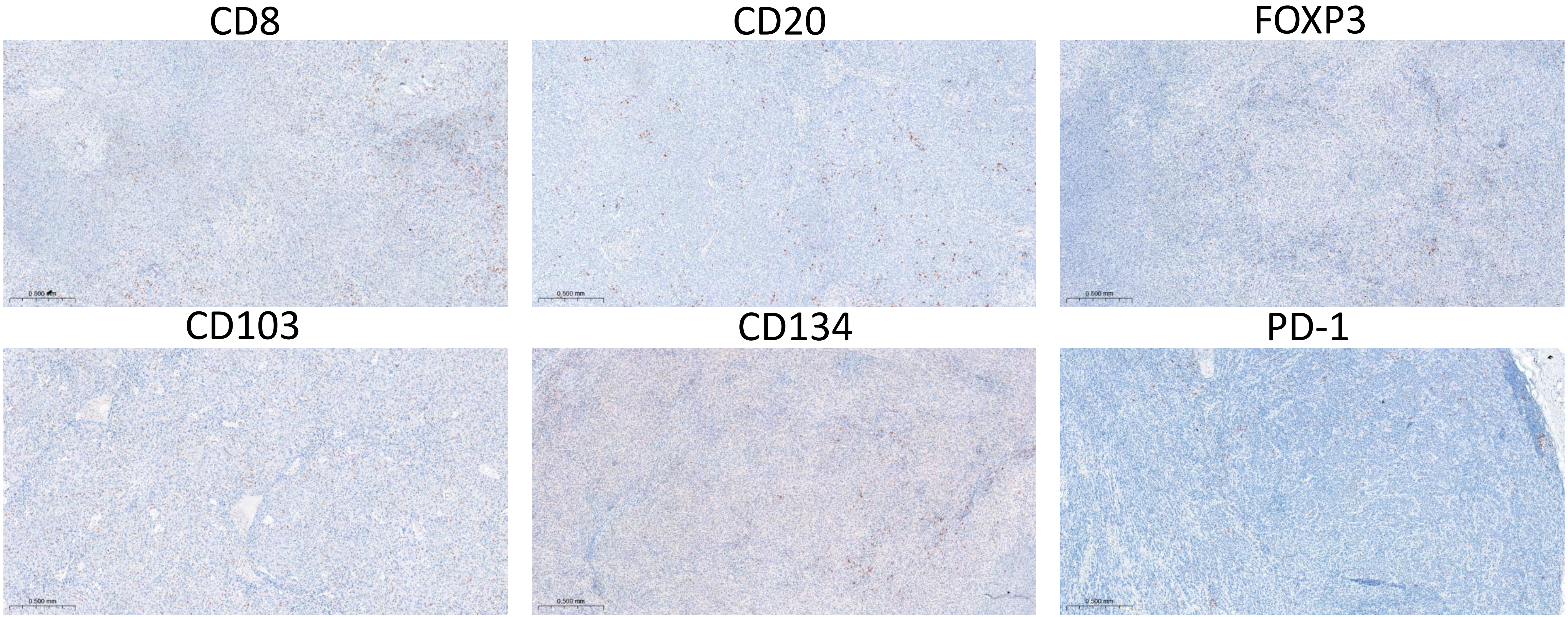

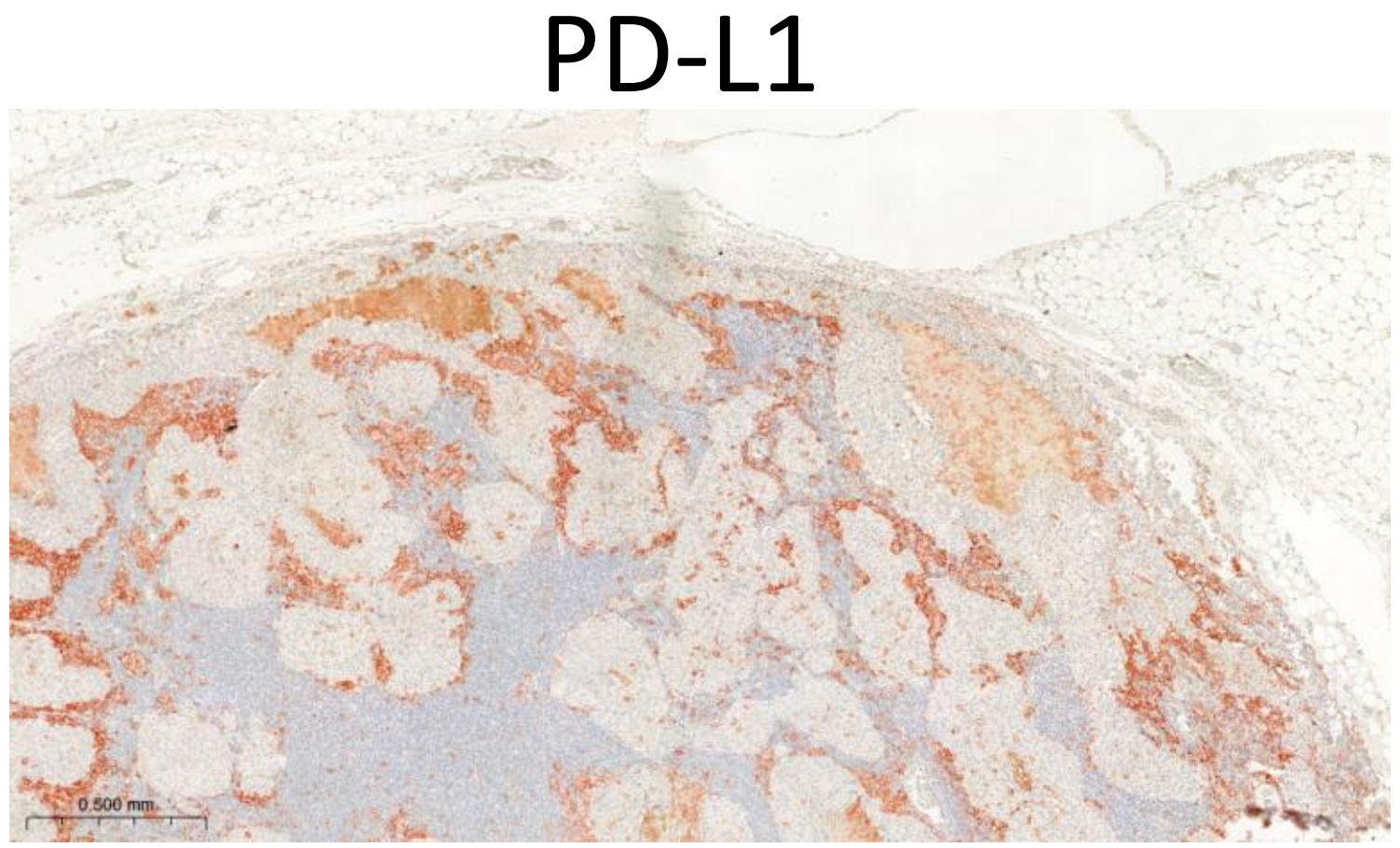

Supplementary Figure 1. Immunohistochemical labeling of immune cell markers in metastatic melanoma samples at low magnification (AEC, red; scale bar: 500 μm)
